# Feeling younger and identifying with older adults: Testing two routes to maintaining well-being in the face of age discrimination

**DOI:** 10.1371/journal.pone.0187805

**Published:** 2017-11-08

**Authors:** Bibiana M. Armenta, Katherine Stroebe, Susanne Scheibe, Tom Postmes, Nico W. Van Yperen

**Affiliations:** Department of Psychology, University of Groningen, Groningen, The Netherlands; Harvard Medical School, UNITED STATES

## Abstract

Integrating the social identity and aging literatures, this work tested the hypothesis that there are two independent, but simultaneous, responses by which adults transitioning into old age can buffer themselves against age discrimination: an individual response, which entails adopting a younger subjective age when facing discrimination, and a collective response, which involves increasing identification with the group of older adults. In three experimental studies with a total number of 488 older adults (50 to 75 years of age), we manipulated age discrimination in a job application scenario and measured the effects of both responses on perceived health and self-esteem. Statistical analyses include individual study results as well as a meta-analysis on the combined results of the three studies. Findings show consistent evidence only for the individual response, which was in turn associated with well-being. Furthermore, challenging previous research, the two responses (adopting a younger subjective age and increasing group identification) were not only theoretically, but also empirically distinct. This research complements prior research by signaling the value of considering both responses to discrimination as complementary rather than mutually exclusive.

## Introduction

Age discrimination against older adults is pervasive and has been shown to negatively affect self-esteem, cognition, behavior, physiological function, health, willingness to live, and even mortality [1–3]. Although there is a wealth of research on responses to discrimination of social groups other than age in the social psychology literature, researchers in this area have paid relatively little attention to *age* discrimination [4]. It thus remains open whether existing knowledge on responses to discrimination can be generalized to older adults. In contrast to other frequently studied stigmatized groups, such as gender or racial groups, age is not a stable characteristic as everybody eventually becomes an ‘older adult’. This leaves us with a lack of knowledge on how older adults respond to age discrimination and which strategies help them to protect their well-being when facing discrimination.

Within the social psychological literature, a large body of research on social identity suggests that older adults might engage in collective responses to discrimination, such as increasing feelings of connection with other older adults upon feeling discriminated against [5–7]. This is based on the assumption that social identities have well-being advantages, even for those who feel disadvantaged due to their group memberships. For example, stronger group identification–via increased feelings of connection with one’s group–can provide support in dealing with experiences of discrimination [8].Yet these studies have largely focused on ethnic minorities and women, not on older persons. Research in the area of aging has almost exclusively focused on responses to older age stereotypes, and rarely on experiences of discrimination (see [9] for an exception). This work suggests that older adults may prefer a more individual response to deal with concerns about older age, for example by considering themselves subjectively younger than their actual age [10,11]. Despite these quite different potential responses to discrimination, no work so far has taken an integrative approach to consider the different ways in which older adults respond to discrimination and how this affects psychological well-being.

The present work integrates the aging and social identity literatures to examine two potential coping responses older targets may follow in response to age discrimination: feeling younger and increasing identification with the group of older people. At first sight, these two responses might seem each other’s opposites: Older adults who feel younger should also identify less with the group of older adults. Accordingly, with few exceptions [12,13], subjective age and group identification have been seen as inverse and interchangeable within aging research [1,14–16]. In this study, we clearly differentiate the two constructs. We assume that feeling younger (individual response) and feeling connected with one’s age group (collective response) may both separately buffer older adults against experiences of discrimination. Therefore, we first test whether the individual and collective responses are not only theoretically, but also empirically distinct concepts. More importantly, we explore the idea that they may be two distinct routes to explain the effects of discrimination on well-being in older adults entering old age. Specifically, we investigate how these routes affect two important well-being outcomes of discrimination: subjective health, considered a main constituent of successful aging [17], which has received little attention in the experimental social psychological literature on discrimination (but see [18]), and self-esteem, a frequently studied outcome of discrimination [19].

### Subjective age and group identification as distinct responses to discrimination

The strategies available to older adults in the face of discrimination may differ from those available to members of more typically studied social groups such as women and ethnic minorities. This is because the group boundaries of the categories of women and ethnicity are typically experienced as clear and undisputed. Indeed, the social psychological literature has mainly considered groups with impermeable boundaries, groups that members cannot leave individually. For this reason *collectively* oriented responses, directed at the group as a whole, have been the main focus of research. Within this approach, the Rejection Identification Model suggests a collectively oriented way of coping with discrimination by which targets increase their levels of identification with their social group as a means of seeking support from other group members ([5,6] but see [20]). Thereby, identifying with the devalued group buffers well-being in the face of discrimination.

Evidence for the Rejection Identification Model has been found among many types of stigmatized groups (e.g., African Americans [5]; women [7]; body piercers [6]). One of the few studies in the area of discrimination that has considered older adults also supported the Rejection Identification Model: older adults showed increased levels of identification with their age group in response to age discrimination, which in turn, alleviated the harmful effects of age discrimination on psychological well-being [20]. Importantly, this work considered adults with a mean age of 75, an age at which boundaries between old and young are relatively clear. It therefore remains open whether identification also buffers against discrimination when group boundaries are more flexible, such as for middle aged adults entering older ages (e.g., [21]).

The fact that boundaries between middle-aged and older adults are flexible–such that it is not clear whether and when to define oneself as an older adult–provides the potential for additional responses to age discrimination. Indeed, older adults have been shown to be flexible in assessments of their own age and whether they “feel old” (e.g., [22,23]). Older adults may be motivated to appear and to feel younger, for example by changing physical appearance via cosmetic surgery and non-surgical cosmetic procedures [11,24], or by construing their subjective age to be younger than their chronological age–a phenomenon referred to as subjective age bias. Subjective age bias is thought to work as a self-enhancing strategy because looking, acting and feeling young is considered to be something positive, at least in Western cultures, and because feeling younger restores feelings of control which are hampered at older ages [11,25].

Interestingly, the gap between subjective and chronological age tends to increase with age [15,22]. Furthermore, subjective age has been shown to vary daily as a function of affective experiences [26]. Moreover, recent research has started to link subjective age bias with experiences of age stigmatization. One large correlational study has revealed a negative, albeit very small relationship (*r* = -.007) between chronic experiences of age discrimination and subjective age bias [9]. However, given the correlational nature of the study data, it is possible that this is because those who ‘feel older’ are more aware of age discrimination, and not necessarily because they objectively experience more discrimination. In contrast, experimental manipulations of exposure to negative stereotypes about aging have been shown to *increase* subjective age bias: Studies that manipulate the salience of stereotypes found that older adults are more likely to feel younger when negative stereotypes of their age are made salient, and to assimilate to pictures of middle-aged as opposed to older adults when receiving negative as opposed to positive or neutral information about their age [10,13]. Note that these prior experimental studies have exposed participants to age stereotyping (the cognitive manifestation of prejudice), not discrimination (the behavioral manifestation of prejudice that concerns personally felt, and experienced, social devaluation [27]). It thus remains open whether increased subjective age bias also occurs in response to experimental manipulations of age discrimination. The present work explores this potential individual level response and assess whether experiences of discrimination also increase subjective age bias (as research on stereotyping suggests), such that greater perceptions of age discrimination are associated with lower subjective age perceptions.

Importantly, subjective age bias also has the potential to benefit psychological well-being and health in the face of discrimination. Notably, experimental research so far has not considered the processes underlying the relation between discrimination and health via subjective age bias. Correlational data has found subjective age bias to be positively correlated with psychological well-being, subjective health, life satisfaction, positive affect, and self-esteem [13,28,29]. Translating these findings to the area of discrimination suggests that: a younger subjective age may boost subjective health and self-esteem in response to discrimination.

### Can individual (subjective age) and collective responses (group identification) coexist?

The considerations presented above suggest that there may be two potential routes by which older adults can respond to age discrimination: a collective route via increased group identification, and an individual route via increased subjective age bias. In the aging literature, these two routes are often conflated as aging researchers tend to use the term “age group identification” to refer to the concept of subjective age. Indeed, in much of the aging literature, *age group identification*—how much older adults identify with the group of older adults—is conceptualized as *subjective age*—how old they feel [1,14–16]. An exemption is the work of Weiss and Lang [12,13], which previously measured age-group identification and subjective age bias as distinct constructs though without distinguishing them at the conceptual level. Nevertheless, the dominant view of age group identification and subjective age bias as each other’s opposites suggests that there should be an inverse relation between the two: The younger older adults feel, the less they identify with older adults. The present work challenges this view by demonstrating the empirical distinctiveness of both concepts, but more importantly, by suggesting that subjective age and age group identification may target qualitatively different types of coping responses–either at the individual or at the collective level.

Indeed, this distinction between individual versus collective level responses is one that is gaining increasing attention within social psychology. Traditionally, individual responses, seeking to personally resolve and/or avoid the disadvantages (e.g., discrimination) associated with one’s group membership, have been seen as mutually exclusive from collective responses. Such collective responses, in which group members seek to collectively resist disadvantage, for example by displaying solidarity or engaging in collective actions to fight stigma within society [30], are thought only to take place when individual responses are not available [31,32]. However, more recently it has been argued that responses which are seen as individually motivated may actually serve the collective [33], and that a strong commitment to the collective need not preclude individual action [34]. Indeed, research on the queen bee effect reveals that women can cope with disadvantage by working at individual advancement in a male dominated environment (an individual response) while at the same time feeling highly connected to and identified with their gender group [35]. This finding dovetails with historical examples of women’s rights movements in which women adopted behaviors of the high status group (e.g., appearing strong, slogans such as ‘we can do it’) yet at the same time remained highly identified with other women.

Furthermore, a correlational study by Weiss and Lang [13] found that feeling younger (individual response) and identifying with the group of older adults (collective response) were negatively associated for adults over the age of 65, but that this relationship was weaker for adults between 40 and 64 years of age and non-significant for younger adults between 18 and 39 years of age. The current study focuses on a more permeable group, that is, older adults in their fifties to seventies. We predict that older adults, especially in the period of transition from midlife to old age, cope with negative societal attitudes and behavior by feeling and acting younger while at the same time feeling identified with the group of older adults.

### Summary of hypotheses

In three studies we examined the proposed alternative routes to maintain well-being in face of age discrimination in the work context. Discrimination against people in their last years of employment (i.e. between 50 and 75 years of age) is well-documented and found to be pervasive, widely legitimated and negatively affecting well-being [36–38]. Therefore, we deemed the work context to be a relevant and representative domain to manipulate age discrimination and test our hypotheses. We predicted that the presence as opposed to absence of age discrimination in a job application scenario strengthens subjective age bias such that older participants feel younger (Hypothesis 1a) and, at the same time, leads to higher older age group identification (Hypothesis 1b). We further predicted that feeling younger (Hypothesis 2a) and identifying more strongly with the older age group (Hypothesis 2b) are both related to higher subjective health and self-esteem. Furthermore, we predicted that both responses mediate the effects of discrimination on well-being, such that the negative effects of age discrimination on subjective health and on self-esteem are diminished through a stronger subjective age bias (Hypothesis 3a) and a stronger identification with the group (Hypothesis 3b).

## Method

Given that the three studies were very similar in design, procedure, and measures, they are described conjointly in one Method section.

### Samples

Participants of all three studies were located in the U.S.A. and were aged 50 to 75 years. We selected people above 50 years of age as the United States anti-age discrimination law protects applicants/employees above age 40 and adults above 50 are considered older adults in organizational settings. Participants were unaware of this age-based selection, those who indicated being of ages between 50 and 75 on an initial demographic survey were invited to participate in the present follow up study. Not knowing the power of the effect a priori, in Study 1 we aimed for, and stopped collecting data, when we reached a sample size of 60 participants per cell. This was based on a rule of thumb that this gives 90% power of detecting a medium size effect (*r* = .30), see Cohen [39], p.384). Post-hoc analyses confirmed that the main results achieved adequate power. According to power analyses based on the results of Study 1 on the two routes, in Study 2 we aimed at 144 participants to achieve 80% power on the main results. According to power analyses based on the results of Studies 1 and 2 on the two routes, in Study 3 we aimed at 100 participants per cell to achieve 80% power on the main results.

Study 1 and Study 2 included 126 and 145 participants, respectively, who were recruited online via Amazon’s Mturk. Mturk or Mechanical Turk is a site from Amazon Web Services that recruits participants around the world to do small jobs through the internet, such as completing questionnaires for businesses or researchers. We ensured that participants of Study 2 had not participated in Study 1 via participants’ Mturk identification numbers. Participants who had participated in Study 1, as identifiable via their ID numbers, were not given access to Study 2. Study 3 included 217 participants recruited online via Qualtrics Panels who was contracted to distribute the survey to the targeted respondents, and to collect the data. Participants of Studies 1 and 2 viewed an advertisement of our study in MTurk’s webpage as a short demographic questionnaire with the possibility of participating in a follow up study about “general experiences” based on their demographics. Following MTurk typical payment rates [40], participants in Studies 1 and 2 received 0.90 dollars for survey completion. Participants in Study 3 received an email invitation of Qualtrics Panels informing them that the survey was for research purposes only, how long the survey was expected to take and what incentives were available. Following Qualtrics Panels’ regulations, remuneration of participants in Study 3 varied depending on the length of survey, panelist profile and acquisition difficulty. The reward type varied and included cash, airline miles, gift cards, redeemable points, sweepstakes entrance and vouchers. Members could unsubscribe at any time.

In Study 1, a total number of 1285 participants replied to the demographic screening survey of which 143 complied with the age requirement. Of these, 11 participants did not complete the survey’s main questions and were excluded from analyses. In Study 2, a total number of 1556 participants replied to the demographic screening survey of which 164 complied with the age requirement. Of these, 11 participants did not complete the survey’s main questions and were excluded from analyses. In Study 3, a total number of 235 participants completed the survey reported in this manuscript (see Design and Procedure for a clarification on this issue) of which 34 did not complete the survey’s main questions and were excluded from analyses. (see Table 1 for more information on the samples).

**Table 1:** Samples composition and participants demographics of Studies 1, 2 and 3.

Study	Sample size	No. Outliers	Mean Age	SD Age	% female	Work status	Level of education	Recruitment date	Drop-out rate
1	126	6^1^	57.3	5.79	66.90%	27% full-time; 20.6% part-time; 22.2% unemployed; 30.2% retired	8.7% high school; 6.3% vocational or technical school; 25.2% some college; 35.4% college degree; 15.7% master’s degree; 3.1% professional degree; 3.1% doctoral degree; 2.5% other	Mar 2013- Jun 2013	7.69%
2	145	8^2^	57.16	5.43	66.20%	38.6% full-time; 22.1% part-time; 20.7% unemployed; 18.6% retired	14.5% high school; 8.3% vocational or technical school; 29.7% some college; 35.9% college degree; 9% master’s degree; 1.4% professional degree; 1.4% doctoral degree	Nov 2013- Jan 2014	6.70%
3	217	17^3^	56.81	5.5	59.40%	58.1% full-time; 19.8% part-time; 22.1% unemployed	29% high school; 9.2% vocational or technical school; 24.4% some college; 26.7% college degree; 8.8% master’s degree; 0.9% professional degree; 0.5% doctoral degree; 0.5% other	Jul-16	14.46%

^1^ Five persons stated that their data should be excluded [41] and one person appeared to be an outlier on the main dependent variable subjective health based on outlier analyses via Cook’s [42] distance (i.e., using the cut-off value of Cook’s distance being larger than four divided by the number of observations).

^2^ Participants appeared to be outliers on the main dependent variable subjective health based on outlier analyses via Cook’s [42] distance.

^3^ Four persons stated that their data should be excluded [41] and thirteen appeared to be outliers on the main dependent variable subjective health based on outlier analyses via Cook’s [42] distance.

### Ethics statement

Before starting the studies a consent form was administered to participants. Participants who did not approve the consent form were not asked to complete the measures. After completion of all measurements, participants were thoroughly debriefed, and were thanked for their participation. Ethical clearance for Study 1, Study 2, and Study 3 was provided by the University of Groningen for research project number ppo-012-114, ppo-013-061, and ppo-015-207, respectively.

### Design and procedure

After providing informed consent, participants were randomly assigned to one of two conditions, discrimination or control, in a between-subjects experimental design. In Study 3 we included one additional manipulation where people either hear that they were rejected for the job (rejection condition) or they do not receive any answer (no rejection condition). The design of Study 3 was therefore a 2 (discrimination vs. no discrimination) by 2 (rejection vs. no rejection) experimental design. To be able to compare results of the three studies, in this manuscript we only report the data of the rejection conditions which are identical across studies. Participants read a hypothetical discrimination scenario adapted from Stroebe and colleagues [21], which is commonly used in the discrimination literature (see also [43,44]. They were asked to imagine that they were taking part in a job selection procedure for a highly appealing job (i.e., in their area of expertise, high-ranking, central to the company, with high income, flexible working hours, and fringe benefits). This was followed by additional information about the interviewer, which differed per condition. In the *discrimination* condition, the interviewer was described as someone who is prejudiced with regard to older people: selecting applicants that were described conform stereotypes of a young person (i.e., “according to him the right candidate should be highly productive, flexible, agile and willing to learn about new technologies”) and selecting more young than old applicants (i.e., “80% younger than 50 years when 50% of the candidates were above 50 years old”). In the *control* condition, the description of the interviewer was neutral: Someone who selects applicants based on their competencies and whose latest selection decisions favored 50% applicants younger than 50 years when 50% of the candidates were above 50 years old. Following this description, participants in both conditions were informed that the interviewer did not consider them a suitable candidate. Afterwards, participants completed the dependent variables and additional measures not included in this report: Emotion regulation strategies, perceived status of older adults (Study 1); emotions, perceived permeability, desired age and longevity (Studies 1 and 2); felt similarity with the group of older adults, legitimacy of treatment (Study 2); stereotypes of older and younger adults (Studies 2 and 3); action intentions, perceived group discrimination, control questions (assessing attention of participants and credibility of the study), additional demographics (Studies 1, 2 and 3).

### Measures

We used identical measures in the three studies to measure the effectiveness of our manipulation as age discrimination and as personal attribution, group identification, subjective age bias, subjective health, and state self-esteem. The items for each of the multi-item measures presented below were averaged into scales for analyses.

#### Manipulation checks (attributions)

Two items based on Schmitt and Branscombe [44] assessed whether participants attributed the outcome of the selection procedure to *age discrimination* (“I would think that the outcome in the selection procedure was due to age discrimination” and “I would think that the outcome in the selection procedure was based on my age”). Two further items based on Schmitt and Branscombe [44] assessed participants’ *personal attribution* (“I would think that the outcome in the selection procedure was due to who I am” and “I would think that the outcome in the selection procedure was due to something about me”). Items were rated on a scale ranging from 1 (*not at all agree*) to 7 (*very much agree*).

#### Group identification

Identification with the group of older adults was assessed via three items adapted from Doosje, Branscombe, Spears, and Manstead [45], e.g., “I identify with the group of older adults”. Items were rated on a scale ranging from 1 (*not at all*) to 7 (*very much*).

#### Subjective age bias

Participants completed the item: “Most of the time, I feel as though I were about age ___”. *Subjective age bias* was calculated by subtracting subjective age from participants’ chronological age (see also [13,29]). Higher values indicate the tendency to feel younger relative to one’s chronological age.

#### Subjective health

Subjective health was measured by aggregating the scores of three items based on Helmer, Barberger-Gateau, Letenneur, and Dartigues [46] and Idler and Benyamini [47], one each referring to *physical health* (“How would you rate your overall physical health at the present time?”), *mental health* (“How would you rate your overall mental health at the present time?”), and *overall health* (“Compared to other people my age, I believe my overall health to be …”). The scale ranged from 1 (*poor*) to 5 (*excellent*).

#### State self-esteem

State self-esteem was measured with Heatherton and Polivy’s [48] seven-item performance state self-esteem subscale (e.g., “I feel confident about my abilities”). Participants were instructed to rate the statements in terms of how true they were at the current moment on a scale ranging from 1 (*not at all*) to 5 (*extremely*).

### Statistical analysis

The Results section presents the results of each individual study as well as a meta-analysis on the combined results. The meta-analysis was computed using the Metafor package (version 1.9–9) in R (version 3.2.4). The most conservative random-effects model was chosen in which the random variance component was determined using restricted maximum likelihood [49]. Effect sizes (standardized regression coefficients β) were calculated for the examined relationships.

## Results

### Preliminary analyses

Reliabilities, means, standard deviations and correlations of central variables are shown in Tables 2–4. There were no differences per condition in age, gender, level of education, or employment status (all *p*’s > .05) and the pattern of results did not change when controlling for these demographic characteristics.

**Table 2:** Reliabilities, means and standard deviations (per condition), and correlations between central study variables in Study 1.

		Cronbach’s α	Control		Discrimination									
			*M*	*SD*	*M*	*SD*	1	2	3	4	5	6	7	8
1	Age	-	57.66^a^	6.42	56.95^a^	5.14	-							
2	Gender^1^	-	64.5%^a^	-	69.2%^a^	-	.10	-						
3	Discrimination Attribution	.97	3.27^a^	2.05	5.45^b^	1.25	.04	.09	-					
4	Personal Attribution	.81	5.31^a^	1.21	4.33^b^	1.67	-.02	-.14	-.34***	-				
5	Group Identification	.84	5.20^a^	1.56	5.86^b^	1.07	.10	.03	.32***	-.10	-			
6	Subjective Age	-	48.29^a^	11.61	42.33^b^	9.65	.39***	.24**	-.07	.12	.21*	-		
7	Subjective Age Bias	-	9.37^a^	10.32	14.08^b^	9.4	.15	-.20*	.12	-.14	-.17	-.85***	-	
8	Subjective Health	.86	3.58^a^	0.99	3.95^b^	0.75	.10	-.12	.19*	-.15	-.13	-.39***	.48***	-
9	State Self-Esteem	.78	4.19^a^	0.66	4.39^a^	0.43	.16	-.19*	.12	-.13	.01	-.16	.28**	.45***

*Notes*. *N* = 126. Gender is coded 1 for female and 2 for male.

* p < .05

** p < .01

*** p < .001.

a,b Means with differing superscripts within rows are significantly different at the p < .05.

^1^Given percentages refer to percentage of female participants on each condition.

**Table 3:** Reliabilities, means and standard deviations (per condition), and correlations between central study variables in Study 2.

			Control		Discrimination									
		Cronbach’s α	*M*	*SD*	*M*	*SD*	1	2	3	4	5	6	7	8
1	Age	-	57.46^a^	5.16	56.84^a^	5.70	-							
2	Gender^1^	-	67.1%^a^	-	65.3%^a^	-	-.01	-						
3	Discrimination Attribution	.97	3.21^a^	1.91	5.08^b^	1.49	.13	-.01	-					
4	Personal Attribution	.80	4.58^a^	1.72	3.81^b^	1.57	-.18*	.01	-.18*	-				
5	Group Identification	.81	5.54^a^	1.42	5.13^a^	1.38	.15	-.05	.06	.12	-			
6	Subjective Age	-	50.25^a^	14.42	43.24^b^	9.34	.26**	.02	-.06	.11	.12	-		
7	Subjective Age Bias	-	7.22^a^	13.98	13.61^b^	9.54	.17*	-.01	.12	-.19*	-.06	-.91***	-	
8	Subjective Health	.82	3.53^a^	0.86	3.86^b^	0.64	.11	-.01	.06	-.24**	-.08	-.35***	.40***	-
9	State Self-Esteem	.76	4.20^a^	0.62	4.29^a^	0.48	.13	.09	-.04	-.24**	.00	-.20*	.26***	.29***

*Notes*. *N* = 145. Gender is coded 1 for female and 2 for male.

* *p* < .05

** *p* < .01

*** *p* < .001.

^a,b^ Means with differing superscripts within rows are significantly different at *p* < .05.

^1^Given percentages refer to percentage of female participants on each condition.

**Table 4:** Reliabilities, means and standard deviations (per condition), and correlations between central study variables in Study 3.

		Cronbach’s α	Control		Discrimination									
			*M*	*SD*	*M*	*SD*	1	2	3	4	5	6	7	8
1	Age	-	57.35^a^	5.84	56.25^a^	5.07	-							
2	Gender^1^	-	58.2%^a^	-	60.7%^a^	0.49	.22**	-						
3	Discrimination Attribution	.91	3.23^a^	1.78	4.57^b^	1.61	.17*	-.05	-					
4	Personal Attribution	.66	4.10^a^	1.53	4.33^a^	1.46	-.04	.02	.22**	-				
5	Group Identification	.73	5.00^a^	1.15	5.04^a^	1.20	.10	.06	.11	.11	-			
6	Subjective Age	-	47.37^a^	9.82	45.80^a^	9.86	.32***	.19**	.04	.12	.14*	-		
7	Subjective Age Bias	-	9.98^a^	9.20	10.49^a^	10.11	.25***	-.08	.06	-.15*	-.08	-.84***	-	
8	Subjective Health	.76	3.72^a^	0.66	3.77^a^	0.67	.24***	.04	.07	-.05	.10	-.11	.25***	-
9	State Self-Esteem	.77	4.05^a^	0.65	4.03^a^	0.62	.06	.00	-.16*	-.21**	.11	-.05	.08	.31***

*Note*. *N* = 217. Gender is coded 1 for female and 2 for male.

* *p* < .05

** *p* < .01

*** *p* < .001.

a,b Means with differing superscripts within rows are significantly different at the *p* < .05.

^1^Given percentages refer to percentage of female participants on each condition.

**Fig 1:**
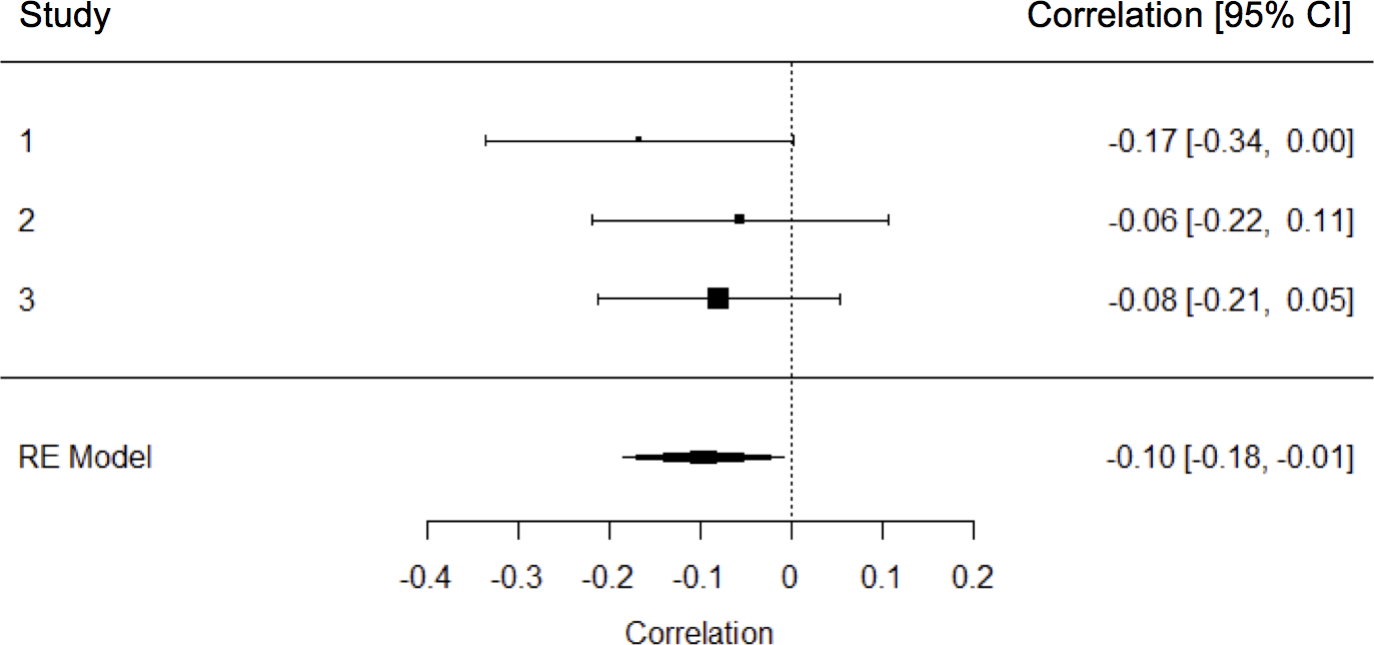
Meta-analytical results of the correlation between subjective age bias and group identification. This forest tree includes the correlation between subjective age bias and group identification of the 3 studies with corresponding 95% confidence intervals in the individual studies. The summary polygon at the bottom of the plot shows the results from a random-effects model when analyzing all 3 studies. RE Model = Random-effects model.

Confirming the independence of the individual and the collective responses to age discrimination, correlations between subjective age bias and group identification were small and ranged from marginal to non-significant (Study 1: *r*(124) = -.17, *p* = .062; Study 2: *r* (143) = -.06, *p* = .497; Study 3: *r* (215) = -.08, *p* = .219). The meta-analysis showed that the combined correlation between subjective age bias and group identification was small and negative with a significant average effect size of β = -0.10, *SE* = 0.04, *Z* = -2.14, *p* = .032, CI [-0.18, -0.01] (see Fig 1).

### Manipulation checks (attributions)

Analyses of variance of the discrimination manipulation on attributions to age discrimination revealed a main effect of condition in all studies: In line with the manipulation, people in the discrimination conditions attributed the outcome of the selection procedure significantly more to age discrimination than people in the control conditions in Study 1, *F*(1, 124) = 53.05, *p* < .001, η^2^_partial_ = .30, Study 2, *F*(1, 143) = 43.11, *p* < .001, η^2^_partial_ = .23, and Study 3, *F*(1, 215) = 33.52, *p* < .001, η^2^_partial_ = .14. Similarly, there was a main effect of condition on personal attributions in two of the three studies: People in the control conditions attributed the outcome of the selection procedure significantly more to the self than people in the discrimination conditions in Study 1, *F*(1, 124) = 14.12, *p* < .001, η^2^_partial_ = .10, and Study 2, *F*(1, 143) = 7.75, *p* = .006, η^2^_partial_ = .05; though not in Study 3, *F*(1, 215) = 1.20, *p* = .274, η^2^_partial_ = .01.

### Hypothesis 1. Effect of condition on subjective age bias and group identification

Our hypotheses are based on the postulate that the condition effects are driven by age discrimination. In order to rule out that condition effects would be driven by the control condition (attributions to the self) rather than the experimental condition (attributions to discrimination), we controlled for personal attributions in all analyses. Note however, that the pattern of results was comparable when attributions were not included. The main results of all hypothesis tests are reported in Table 5. Results of Studies 1 and 2 (but not 3) confirmed Hypothesis 1a: participants in the discrimination condition reported higher subjective age bias than those in the control condition. These effects were confirmed by the meta-analysis which revealed a significant overall effect across studies (β = 0.15).

**Table 5:** Statistical tests of main results in Studies 1 to 3 and meta-analysis of the combined effects of all three studies.

Result tested	Study	Effect size (SE)	Test	p-value	95% CI [LL, UL]
Discrimination → SAB (Hypothesis 1a)	1	*b* = 4.25 (1.94)	*t* (123) = 2.19	0.030	[0.41, 8.09]
	2	*b* = 5.60 (1.91)	*t* (142) = 2.94	0.004	[1.83, 9.37]
	3	*b* = 0.72 (1.31)	*t* (213) = 0.55	0.581	[-1.85, 3.30]
	meta-analysis	β = 0.15 (0.07)	*Z* = 2.27	0.023	[0.02, 0.30]
Discrimination → GI (Hypothesis 1b)	1	*b* = 0.65 (0.25)	*t* (123) = 2.59	0.011	[0.12, 1.18]
	2	*b* = -0.34 (0.24)	*t* (142) = -1.46	0.148	[-0.81, 0.12]
	3	*b* = 0.02 (0.16)	*t* (213) = 0.10	0.922	[-0.30, 0.33]
	meta-analysis	β = -0.03 (0.17)	*Z* = -0.21	0.837	[-0.37, 0.30]
Discrimination → SH (total effect)	1	*b* = 0.32 (0.17)	*t* (123) = 1.97	0.052	[-0.00, 0.65]
	2	*b* = 0.25 (0.13)	*t* (142) = 1.92	0.057	[-0.00, 0.65]
	3	*b* = 0.06 (0.09)	*t* (213) = 0.71	0.481	[-0.12, 0.24]
	meta-analysis	β = 0.12 (.05)	*Z* = 2.43	0.015	[0.02, 0.21]
Discrimination → SSE (total effect)	1	*b* = 0.17 (0.11)	*t* (123) = 1.57	0.119	[-0.04, 0.38]
	2	*b* = 0.03 (0.09)	*t* (142) = 0.31	0.753	[-0.15, 0.21]
	3	*b* = 0.01 (0.09)	*t* (213) = 0.12	0.903	[-0.16, 0.18]
	meta-analysis	β = 0.05 (0.05)	*Z* = 1.12	0.263	[-0.04, 0.14]
SAB → SH (controlling for discrimination, group identification and personal attributions; Hypothesis 2a)	1	*b* = 0.04 (0.01)	*t* (121) = 4.22	< .001	[0.02, 0.05]
	2	*b* = 0.02 (0.00)	*t* (140) = 4.82	< .001	[0.01, 0.03]
	3	*b* = 0.02 (0.01)	*t* (211) = 3.45	< .001	[0.01, 0.03]
	meta-analysis	β = 0.34 (0.05)	*Z* = 6.32	< .001	[0.23, 0.44]
SAB → SSE (controlling for discrimination, group identification and personal attributions; Hypothesis 2a)	1	*b* = 0.01 (0.01)	*t* (121) = 2.45	0.016	[0.00, 0.03]
	2	*b* = 0.01 (0.00)	*t* (140) = 2.73	0.007	[0.00, 0.02]
	3	*b* = 0.00 (0.01)	*t* (212) = 0.67	0.503	[-0.01, 0.01]
	meta-analysis	β = 0.18 (0.06)	*Z* = 2.98	0.003	[0.06, 0.29]
GI → SH (controlling for discrimination, subjective age bias and personal attributions; Hypothesis 2b)	1	*b* = -0.06 (0.06)	*t* (121) = -1.16	0.248	[-0.17, .05]
	2	*b* = -0.01 (0.05)	*t* (141) = -0.32	0.752	[-0.11, 0.08]
	3	*b* = 0.07 (0.04)	*t* (211) = 1.74	0.083	[-0.01, 0.15]
	meta-analysis	β = 0.00 (0.06)	*Z* = 0.07	0.939	[-0.12, 0.13]
GI → SSE (controlling for discrimination, subjective age bias and personal attributions; Hypothesis 2b)	1	*b* = 0.00 (0.04)	*t* (121) = 0.11	0.910	[-0.07, 0.08]
	2	*b* = 0.01 (0.03)	*t* (140) = 0.47	0.642	[-0.05, 0.07]
	3	*b* = 0.07 (0.04)	*t* (211) = 1.68	0.095	[-0.01, 0.16]
	meta-analysis	β = 0.07 (0.05)	*Z* = 1.64	0.101	[-0.01, 0.16]
Discrimination → SAB → SH (controlling for group identification and personal attributions; Hypothesis 3a)	1	*b* = 0.16 (0.08)	*Z* = 1.90	0.057	[0.03, 0.36]
	2	*b* = 0.12 (0.05)	*Z* = 2.49	0.014	[0.05, 0.23]
	3	*b* = 0.01 (0.02)	*Z* = 0.52	0.600	[-0.03, 0.07]
	meta-analysis	β = 0.05 (0.05)	*Z* = 1.12	0.263	[-0.04, 0.14]
Discrimination → SAB → SSE (controlling for group identification and personal attributions; Hypothesis 3a)	1	*b* = 0.06 (0.03)	*Z* = 1.56	0.118	[0.01, 0.15]
	2	*b* = 0.06 (0.03)	*Z* = 1.94	0.053	[0.01, 0.13]
	3	*b* = 0.00 (0.01)	*Z* = 0.31	0.755	[-0.01, 0.04]
	meta-analysis	β = 0.03 (0.05)	*Z* = 0.66	0.506	[-0.06, 0.12]
Discrimination → GI → SH (controlling for subjective age bias and personal attributions; Hypothesis 3b)	1	*b* = -0.04 (0.04)	*Z* = -0.98	0.326	[-0.16, 0.01]
	2	*b* = 0.01 (0.02)	*Z* = 0.26	0.798	[-0.02, 0.06]
	3	*b* = 0.00 (0.01)	*Z* = 0.08	0.933	[-0.02, 0.03]
	meta-analysis	β = -0.00 (0.05)	*Z* = -0.11	0.916	[-0.09, 0.08]
Discrimination → GI → SSE (controlling for subjective age bias and personal attributions; Hypothesis 3b)	1	*b* = 0.00 (0.03)	*Z* = 0.10	0.917	[-0.05, 0.06]
	2	*b* = -0.00 (0.01)	*Z* = -0.37	0.710	[-0.04, 0.01]
	3	*b* = 0.00 (0.01)	*Z* = 0.08	0.933	[-0.02, 0.04]
	meta-analysis	β = -0.00 (0.05)	*Z* = -0.06	0.955	[-0.09, 0 .09]

*Note*. SAB = Subjective age bias; GI = Group identification; SH = Subjective health; SSE = State Self-esteem.

In weak support for Hypothesis 1b (see Table 5) participants in the discrimination condition reported higher group identification than those in the control condition in Study 1, but not in Studies 2 or 3. The meta-analysis revealed a non-significant overall effect across studies (β = -0.03).

### Hypothesis 2. Effects of subjective age bias and group identification on well-being

Confirming Hypothesis 2a, subjective age bias was positively related to subjective health in all three studies (when controlling for discrimination, group identification and personal attribution) which resulted in a significant combined effect across studies (β = 0.34, see Table 5). Moreover, analyses showed that subjective age bias was related to higher state self-esteem (when controlling for discrimination, group identification and personal attributions) in Study 1 and in Study 2 but not in Study 3. Nevertheless, the meta-analysis revealed a significant combined effect across studies (β = 0.18, see Table 5).

In contrast with Hypothesis 2b, analyses showed that group identification was unrelated to subjective health and self-esteem (when controlling for discrimination, subjective age bias and personal attributions) in all three studies, which resulted in non-significant combined effects across studies (β = 0.00 and β = 0.07, respectively, see Table 5).

### Hypothesis 3. Effects of the two routes on well-being

In order to test the mediational role of subjective age bias and group identification in the relationship between discrimination and well-being, we applied Preacher and Hayes’ [50] approach for estimating indirect effects in simple mediation models ([51] Model 4). We ran separate mediation models for the two outcome measures, subjective health and state self-esteem. The models included discrimination as predictor, subjective age bias and group identification entered simultaneously as mediators, and personal attribution as covariate. We requested a 95% bias-corrected interval based on 5000 bootstrap samples.

In partial support for Hypothesis 3a, Studies 1 and 2 (but not 3) provided support for a positive indirect effect of age discrimination on subjective health and on self-esteem through subjective age bias. However, the meta-analysis revealed non-significant overall effects across studies (β = 0.05 and β = 0.03, respectively, see Table 5).

In contrast with Hypothesis 3b, there were no significant indirect effects of age discrimination on subjective health or on self-esteem through group identification in any of the three studies. Obviously, this resulted in non-significant combined effects across studies (β = -0.00, for both outcomes see Table 5).

**Fig 2:**
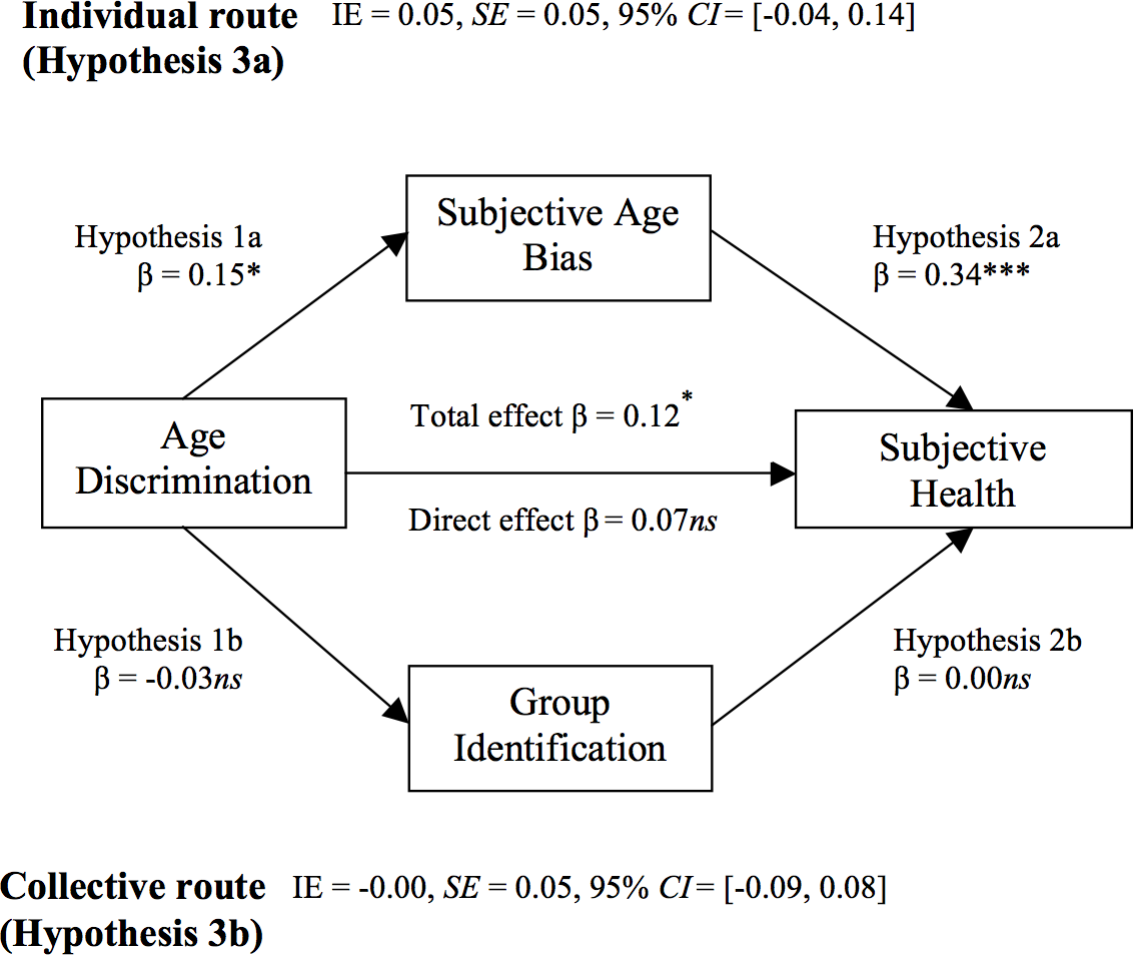
Combined results of the collective and the individual routes on subjective health. Estimates are presented in standardized values (β), after partializing the effects of all other relevant variables. IE = indirect effect of independent variable on dependent variable through the proposed mediator. * p < .05; ** p < .01; *** p < .001; ns: non-significant at the .05 level.

**Fig 3:**
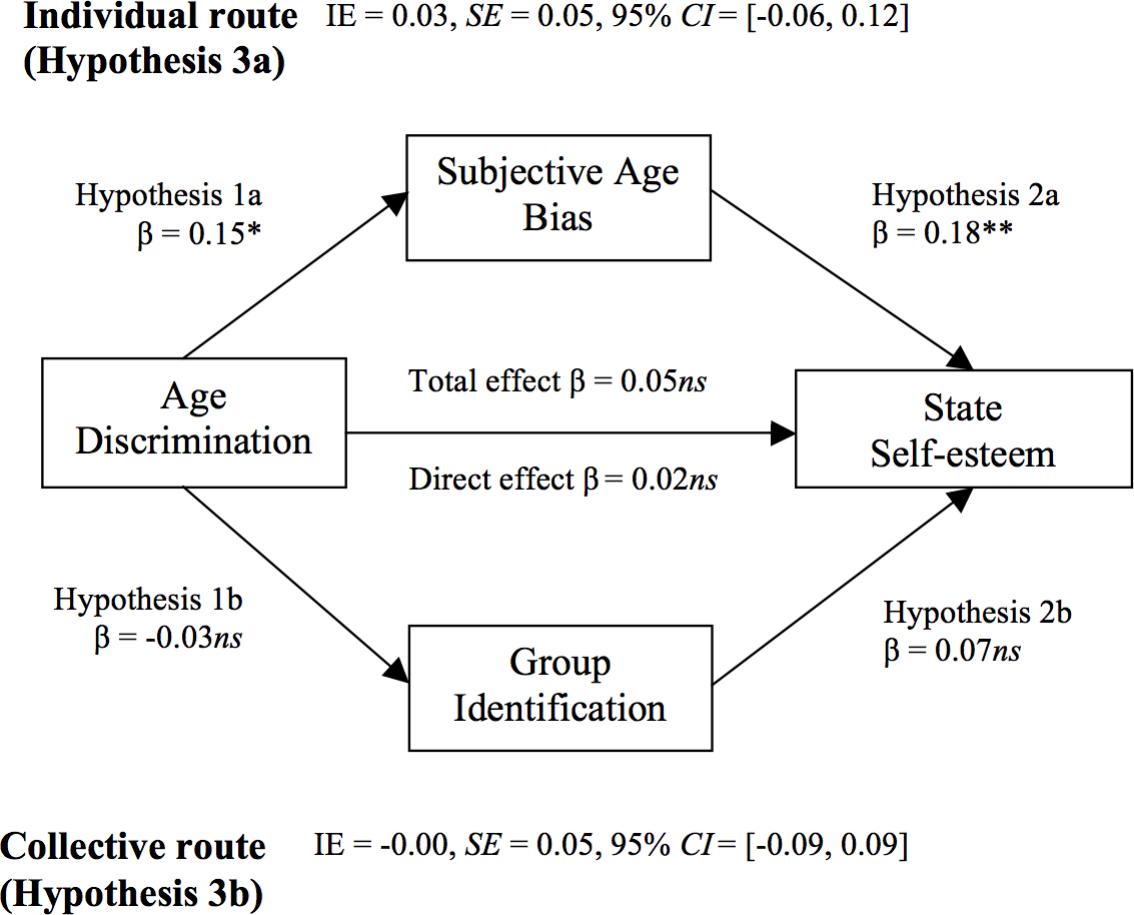
Combined results of the collective and the individual routes on state self-esteem. Estimates are presented in standardized values (β), after partializing the effects of all other relevant variables. IE = indirect effect of independent variable on dependent variable through the proposed mediator. * p < .05; ** p < .01; *** p < .001; ns: non-significant at the .05 level.

The meta-analytical results are summarized and visualized in Fig 2 (Subjective Health) and Fig 3 (State Self-Esteem).

## Discussion

How do older adults buffer themselves against the adverse effects of discrimination? This question has received surprisingly little attention within the social psychological and aging literatures. Whereas the social psychology literature has mainly attended to discrimination of social groups other than age, the aging literature has focused on responses to age stereotypes but not discriminatory behavior. Integrating these literatures, we proposed two parallel routes by which older adults might buffer themselves against experiences of age discrimination: An individual route in which experiences of discrimination are countered by lowering one’s felt, compared to actual, chronological age, and a collective route by which targets turn to their group and increase levels of identification with other older adults.

### Evidence only for the individual route

Previous aging research has focused on the effects of negative stereotyping rather than age discrimination on people’s coping responses and well-being [13,52]. However, negative stereotyping and felt discrimination are two distinct phenomena: Discrimination refers to personally felt social devaluation—thus a behavioral manifestation of prejudice—while stereotyping refers to internalized views of the group—thus a cognitive manifestation [27]. The current findings provided support for the individual route by showing for the first time that older adults respond not only to stereotyping, as previously studied, but also to age discrimination by indicating they feel younger. Furthermore, this response may serve to buffer targets against age discrimination as increases in subjective age bias in response to discrimination were related to improved well-being. Regarding this buffering effect, we note that although the separate effects were significant across studies, we only found indirect effects suggestive of such a buffering process, for Studies 1 and 2. This is not entirely surprising as research indicates that mixed results are highly likely to be encountered when multiple studies are performed [53]. Yet, possible reasons for this divergence in findings are discussed below in the section on limitations. While additional research in this direction is needed, the present results point to the value of considering the individual route, via subjective age, as a viable and important response to age discrimination.

Findings showed less support for a collective route as age discrimination only affected levels of identification in Study 1. In addition, there was no evidence of a buffering effect of age identification on well-being as there was no relation between identification and subjective health and state self-esteem across studies. This may seem surprising in light of the findings of Garstka and colleagues [20] who did provide support for this buffering effect in older adults. Yet the group of adults in the Garstka study sample was older, with a mean age of 75 compared to 57 in our sample. Arguably this is an age at which the boundaries between old and young may be perceived as more clear-cut than in our sample, making the study more similar to work looking at responses to racial and gender discrimination. Future work should consider different age groups that are classified as ‘older adults’ in more detail in order to determine whether indeed the extent to which group boundaries are perceived as set versus more flexible, affect preferences for individual versus collective strategies.

### Subjective age bias and group identification as distinct constructs

One key assumption in the present work was that subjective age bias and group identification concern separate concepts and constitute different routes to buffering well-being. This idea is in contrast to most of the aging literature that thus far has predominantly considered these concepts as interchangeable. The present work underscores that subjective age bias and identification are not interchangeable, at least in the early stages of old adulthood. Subjective age bias and group identification appeared to be not only theoretically, but also empirically distinct constructs. Furthermore, they were differentially associated with well-being outcomes: Whereas group identification was uncorrelated with well-being across studies, subjective age bias consistently correlated with subjective health and self-esteem across studies. This qualifies the traditional view in the aging literature of subjective age as an indicator of older adults’ awareness of their own aging *or* of reduced identification with their age group (cf. [15,54]).

These findings are important because they underscore that group identification and subjective age should not be conflated. Recognizing their differences and studying them separately opens the possibility to incorporate into the aging literature the effects of group identification as largely explored by the social identity tradition. For example, while subjective age may bring about individual types of strategies to cope with disadvantage such as trying to remain younger by doing exercise, dying one’s hair, or training memory skills [11], group identification can bring about collective types of strategies such as participating in a demonstration to stop the discrimination of the ingroup [55]. Furthermore, a conflation of these two constructs may lead researchers to make faulty predictions. For example, while an induced lower subjective age was shown to increase people’s physical strength [56], it is unlikely that group identification decreases physical strength. Or as the results of the present studies showed, while subjective age was associated with higher subjective health and self esteem, group identification was not.

From a social identity perspective these findings are important as researchers often assume that group members face a choice between individual or collective responses, implying that these strategies would be mutually exclusive [30]. Yet more recent conceptual and empirical approaches suggest that an individual response, such as being individually mobile within an organization, need not preclude a collective response, such as identifying with and supporting members of one’s group [33,57]. The present research provides further evidence in this direction, suggesting the importance of considering individual strategies as a viable response to discrimination that may also buffer targets against discrimination, while not necessarily undermining loyalty towards one’s group. For example, subjective age bias can boost well-being and thus provide resources at the individual level which may be essential for a collective response (e.g., protesting one’s discrimination). Group identification may serve a more collective need, the desire to address the disadvantaged position of one’s group. Indeed, it has consistently been shown to be an important instigator of collective protest (e.g., [58]). Consequently, the combination of individual resources (feeling young) and group identification (feeling connected with other older adults) may potentially serve to instigate collective responses such as confronting age discrimination against one’s group.

In studying the interplay between individual versus collective strategies, an interesting avenue for future research is the comparability across groups. It is often assumed that collective strategies (e.g, group identification) are the most beneficial for well-being (e.g., [5,19]). Our work speaks to the importance of individual strategies as a way of dealing with discrimination for those who are entering old age. We would argue this also points to the importance of considering how different strategies may befit different groups. In line with this reasoning, a recent meta-analysis of effects of discrimination on well-being revealed that gender and racial discrimination have less negative effects on well-being than many other types of discrimination, such as sexual orientation or disability [19]. Yet at present it is not clear which aspect of these group differences (e.g., concealability, controllability) can explain these differences in well-being [19]. We believe that in understanding group differences in well-being, it may be important to study differences in individual versus collective responses to discrimination between groups.

### Implications for well-being

As already discussed, findings of the present work suggest that feeling younger is beneficial for older adults’ well-being when facing stigma, at least in the short term. A potential intervention could thus be the induction of a younger subjective age when people feel discriminated because of their age. Previous research has shown that inducing a younger subjective age can be achieved through downward social comparisons with people of same age [56]. However, more research is needed to clarify the suitability of such an intervention as we do not fully understand under which conditions a lower subjective age bias is beneficial. For example, we do not know if it is beneficial for adults above our sample’s ages, or for chronic forms of discrimination, nor do we know its longer term benefits.

Furthermore, our findings suggest that group identification is not a route which is consistently followed in face of discrimination and that it is not associated with well-being. However, this does not mean that group identification cannot have beneficial properties. In fact, numerous studies have found evidence for indirect positive well-being effects of group identification through the promotion of attitudes and behaviors that counteract the negative effects of stigma [8,59–61]. Overall, we note that our findings are based on a short-term response to an instance of discrimination. Future research should study more long term effects of individual (subjective age bias) and collective (group identification) effects on well-being.

### Limitations

The present work has some limitations. The studies make use of scenarios to induce discrimination, which are often criticized for having low ecological validity. However, for ethical and practical reasons, scenario methods are often the most feasible way to study the effects of discrimination. Accordingly, the discrimination literature often makes use of scenarios to study effects of discrimination experimentally and these studies reveal results comparable to ‘non-scenario’ studies [43,44,62, 63]. In further favor of the validity of the used scenario, participants in Studies 1 and 3 could very well imagine experiencing the proposed situation (Study 1: *M* = 5.76, *SD* = 1.21; Study 3: *M* = 5.03, *SD* = 1.39; scale from 1 = *very difficult to imagine* to 7 = *very easy to imagine*), and found the scenario believable (Study 1: *M* = 5.73, *SD* = 1.38; Study 3: *M* = 5.34, *SD* = 1.35; scale from 1 = *not at all believable* to 7 = *very believable*; questions not assessed in Study 2).

A second potential limitation pertains to the sample. Recruitment of participants via an Internet site raises concerns regarding the lack of control over respondents, for example, whether they focus on the task or whether they take the task seriously. However, research on Amazon’s Mechanical Turk as a source of data for psychological research showed that these concerns are not substantiated [64,65]. These studies showed that the data obtained via Mturk is at least as reliable as that obtained via traditional methods, a result that is confirmed by the adequate reliabilities found in the present studies (α = .76 - .97). Furthermore, Mechanical Turk has the advantage that participants are more demographically diverse and more representative of the American population than is the case for more traditional recruitment methods [64,65]. In order to avoid relying on a single data source, however, we utilized a different recruitment method in Study 3. In this study we made use of an existing panel of a contracted recruitment agency Qualtrics Panels. Data of Study 3 also showed adequate reliability (α = .66 - .91).

Related to the recruitment of participants via the Internet we note that our sample was familiar with the use of online technologies and were rather highly educated. Yet, importantly, reported levels of perceived personal discrimination indicated that they had experienced age discrimination in the past (Study 2: *M* = 4.14, *SD* = 1.72; Study 3: *M* = 3.54, *SD* = 1.92; scale from 1 = *not at all felt age discrimination* to 7 = *very much felt age discrimination;* questions not assessed in Study 1). Given the composition of our sample, we cannot directly speak to how less educated or less technically literate participants would respond regarding experiences of and responses to age discrimination. Indeed, this group may experience age discrimination differently and for different reasons (e.g., not being technically literate). For those who wish to study in more detail the nature of discrimination against lower educated or less technical older adults we would advise different sampling procedures (e.g., paper and pencil, interviews).

Notably, results of Studies 1 and 2 but not of Study 3 were supportive of subjective age bias as a buffer for well-being when facing age discrimination. In trying to explain this disparity, we reviewed differences between the studies. We found differences in the types of attributions participants made across studies. In Study 3 participants attributed their failure in the selection procedure to themselves to an equal extent, regardless of whether they had experienced discrimination or not. In Studies 1 and 2, self-attributions were lower in the discrimination conditions. Yet controlling for personal attributions did not change the nature of the results. Another difference was that compared to the other two studies, Study 3 did not include retired participants. It is plausible that retired people perceive the boundaries of the group as less permeable and thus for them a collective response may be more viable than an individual response. Indeed, Study 1 which had the highest proportion of retired participants was the only study in support of the collective response. This, as discussed earlier, speaks to the importance of considering the interplay between individual and collective responses to discrimination across different types of groups (see also [66]).

### Conclusion

Findings of the present studies offer support for the idea that an individual response (subjective age bias) and a collective response (group identification) to age discrimination are not mutually exclusive for adults transitioning from midlife to old adulthood. However, findings suggest that feeling younger but not identifying with the group is the preferred response to discrimination in this life period. Furthermore, findings suggest that only feeling younger may boost self-esteem and increase levels of perceived health, while identifying with the group lacks these benefits. This research complements prior research by pointing to the value of considering individual and collective responses to age discrimination as complementary rather than mutually exclusive.

## Supporting information

S1 Data Data of Experiment 1. (SAV)

S2 Data Data of Experiment 2. (SAV)

S3 Data Data of Experiment 3. (SAV)
